# Surveillance colonoscopy findings in asymptomatic participants over 75 years of age

**DOI:** 10.1002/jgh3.13071

**Published:** 2024-05-01

**Authors:** Madelyn Agaciak, Molla M Wassie, Kalindra Simpson, Charles Cock, Peter Bampton, Robert Fraser, Erin L Symonds

**Affiliations:** ^1^ Department of Medicine, College of Medicine and Public Health Flinders University Bedford Park South Australia Australia; ^2^ Flinders University, College of Medicine and Public Health Flinders Health and Medical Research Institute, Adelaide Bedford Park South Australia Australia; ^3^ Department of Gastroenterology and Hepatology Flinders Medical Centre Bedford Park South Australia Australia

**Keywords:** adenoma, aged, colonoscopy, colorectal cancer, colorectal neoplasm, surveillance

## Abstract

**Background and Aim:**

Surveillance colonoscopy for colorectal cancer (CRC) is generally not recommended beyond 75 years of age. The study determined incidence and predictors of advanced adenoma and CRC in older individuals undergoing surveillance colonoscopy.

**Methods:**

This was a retrospective cohort study of asymptomatic older participants (≥75 years), enrolled in a South Australian CRC surveillance program who underwent colonoscopy (2015–2020). Clinical records were extracted for demographics, personal or family history of CRC, comorbidities, polypharmacy, and colonoscopy findings. The associations between clinical variables and advanced adenoma or CRC at surveillance were assessed with multivariable Poisson regression analysis.

**Results:**

Totally 698 surveillance colonoscopies were analyzed from 574 participants aged 75–91 years (55.6% male). The incidence of CRC was 1.6% (11/698), while 37.9% (260/698) of procedures had advanced adenoma detected. Previous CRC (incidence rate ratio [IRR] 5.9, 95% CI 1.5–22.5), age ≥85 years (IRR 5.8, 95% CI 1.6–20.1) and active smoking (IRR 4.9, 95% CI 1.0–24.4) were independently associated with CRC diagnosis, while advanced adenoma at immediately preceding colonoscopy (IRR 1.6, 95% CI 1.3–2.0) and polypharmacy (IRR 1.2, 95% CI 1.0–1.5) were associated with advanced adenoma at surveillance colonoscopy in asymptomatic older participants (≥75 years).

**Conclusion:**

Advanced neoplasia was found in more than one third of the surveillance procedures completed in this cohort. Continuation of surveillance beyond age 75 yeasrs may be considered in participants who have previous CRC or are active smokers (provided they are fit to undergo colonoscopy). In other cases, such as past advanced adenoma only, the need for ongoing surveillance should be considered alongside participant preference and health status.

## Introduction

Colorectal cancer (CRC) is one of the most common cancers worldwide,^1^ and its incidence increases with advancing age.^2^ As the number of individuals aged older than 80 years is projected to increase more than threefold over the next 30 years,^3^ appropriate CRC prevention strategies are required. While most secondary prevention programs for screening and surveillance cover ages up to 75 years of age, it remains unclear when to stop these programs in older cohorts.^4^

The majority of CRC develop via an adenoma to carcinoma pathway,^5^ and early detection and removal of adenomas or other pre‐cancerous neoplastic lesions prevents transformation into cancer, reducing CRC incidence over time.^6^ Many countries utilize fecal occult blood tests (FOBT) within organized screening programs to aid the early detection of colorectal neoplasia,^7^ while regular surveillance with colonoscopy is recommended in participants with a prior history of neoplasia, or significant family history of CRC.^8^

While there are proven benefits in screening and surveillance through decreased incidence and mortality from CRC,^9^, ^10^, ^11^ the decision for continuing surveillance in individuals over 75 years is complex, and the guidelines related to this vary worldwide. The American Society for Gastrointestinal Endoscopy (ASGE) avoids providing recommendations for surveillance in participants ≥75 years due to uncertainty in utility.^12^ The European Society of Gastrointestinal Endoscopy (ESGE) Guidelines provide a weak recommendation for surveillance to cease at age 80 years, or earlier if comorbid conditions limit life expectancy.^13^ Current Australian guidelines state that surveillance will not benefit most people over 75 years, instead advising individualized clinical recommendations.^14^ Many studies assessing colonoscopy in older individuals are limited to a screening context, with a recent study supporting that the risk of mortality from other causes is greater than that from CRC.^15^ Overall, there are limited data on the benefits of surveillance colonoscopy beyond the age of 75 years, particularly where other risk factors are considered. For individuals and clinicians to make informed decisions about continuing surveillance colonoscopy beyond 75 years, it is important to determine risk factors, beyond age alone, associated with the findings of CRC and advanced adenoma in this population, particularly given the increased frequency of comorbidities and procedural complications, and reduced life expectancy.^16^ Moreover, the incidence of CRC and advanced adenoma is not previously well documented in older people at above average risk of CRC due to personal history of neoplasia or family history of CRC.

This study aimed to determine the incidence of CRC and advanced adenoma during colonoscopies in asymptomatic surveillance participants over 75 years of age, as well as to investigate the demographic and clinical risk factors associated with developing these advanced neoplastic lesions.

## Methods

### Setting, study design, and population

This was a retrospective analysis (2015–2020) in participants aged 75 years and older at the time of surveillance colonoscopy, and who were all enrolled within the South Australian Southern Cooperative Program for the Prevention of Colorectal Cancer (SCOOP; a surveillance colonoscopy program for individuals with a prior history of colorectal neoplasia or a significant family history of CRC).^17^, ^18^, ^19^ Colonoscopies were performed at three public hospitals in the southern regions of Adelaide (Flinders Medical Centre, Noarlunga Hospital, Repatriation General Hospital). In this population, colonoscopy undertaken for surveillance purposes in asymptomatic participants, with pathology results available, were included. Colonoscopy performed for other indications, or those with a poor bowel preparation or incomplete intubation distance (not reaching the cecum), were excluded from analysis. In addition, cases were excluded when pathology outcomes were indeterminate, or with a diagnosis of inflammatory bowel disease.

### Assessment of risk factors

For each colonoscopy, clinical records documented at the time of colonoscopy were reviewed, and age, sex, body mass index (BMI‐ kg/m^2^), and socioeconomic status by residential area^20^ recorded. Age was analyzed as categorical variable (75–79.9, 80–84.9, and ≥85 years). BMI was assessed as a continuous variable The residential area postcode enabled calculation of relative socioeconomic advantage and disadvantage information (a higher score indicates a relative lack of disadvantage).^20^ Socioeconomic position was divided into two categories—those above and below the median score for relative socioeconomic advantage and disadvantage.

Medical comorbidities, presence of polypharmacy (≥5 individual medications),^21^ smoking status (nonsmoker, ex‐smoker, and active smoker) and history of alcohol intake (nondrinkers and drinkers [social drinkers as well as 1 or more standard drink per day]) were also recorded. Medical comorbidities were translated to the modified Charlson Comorbidity Index (CCI), rather than the classical CCI,^22^ where an increasing CCI indicates a decreased likelihood of 10‐year survival. Personal and family history of CRC was documented as binary categories (yes *vs* no).

### Assessment of outcomes

Pathology findings were assessed and grouped as: (i) CRC – cancer with invasion beyond the muscularis mucosa; or (ii) advanced adenoma – including adenomas and sessile serrated lesions ≥10 mm, adenomas with high grade dysplasia or villous changes, and sessile serrated lesions with any cytological dysplasia. In addition, ≥3 tubular adenomas (<10 mm), as well as concurrent small tubular adenomas and sessile serrated lesions (of any size) were included in the advanced definition. This was because at the time of most of the procedures in the audit, the Australian guidelines recommended a subsequent surveillance interval of a 3‐years for these findings, similar to that for other forms of advanced adenoma.^23^ The term advanced neoplasia was inclusive of all advanced adenoma or CRC. Staging of CRC was based on the American Joint Committee on Cancer staging (AJCC guidelines version 8).^24^ All other pathology findings at surveillance colonoscopy were grouped as non‐advanced.

### Data analysis

Descriptive statistics were calculated to establish participants demographics, including median with interquartile range (IQR). The analyses were performed at two levels: firstly, analysis of the whole cohort, and secondly after stratification based on a previous history of CRC (at any time during life; “Lifetime CRC”). Chi‐squared tests were used to compare the incidence of advanced adenoma and CRC. The Kruskal Wallis test was used to compare the median of continuous data between groups (including age and number of previous colonoscopies). The extent of missing data in this study varied between 2% (for CCI) and 37% (for alcohol intake). Consequently, multiple imputation employing chained equations was employed to address missingness while assessing relevant predictors of advanced adenoma or CRC. The missing data pattern exhibited non‐monotonic behavior, and it was assumed that the data were missing at random.^25^ A total of 20 imputed datasets were created. The incomplete variables included in the imputation process were BMI, smoking status, number of medications, alcohol intake, and CCI. Multivariable Poisson regression analysis was undertaken to determine variables associated with advanced adenoma or CRC findings at surveillance. Expert opinion and evidence from literature were used to identify the risk factors of CRC or advanced adenoma that were included in the regression models. Few variables such as number of prior colonoscopies were excluded in the regression models due to multicollinearity. Incidence Rate Ratio (IRR) with 95% confidence intervals (CI) were reported. The multivariable analyses were performed on the imputed dataset adjusting for the key demographic and clinical predictors for CRC as well as for advanced adenoma. All analyses were performed using STATA version 16.0 (StataCorp, TX, USA), with a *P* value <0.05 considered statistically significant.

## Results

**Figure 1:**
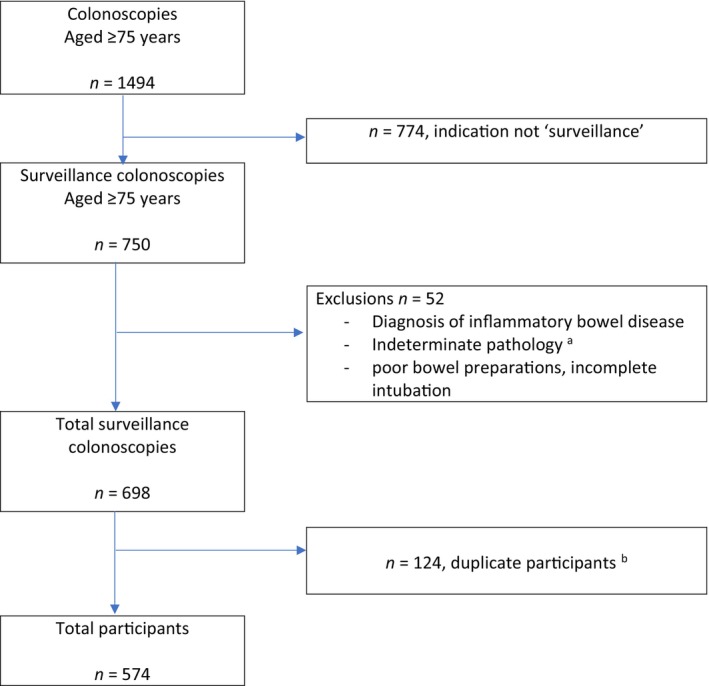
Flow diagram of colonoscopies performed in participants aged ≥75 years enrolled in a colonoscopy surveillance program (2015–2020), due to prior history of neoplasia or significant family history of colorectal cancer. ^a^Any pathology finding that could not be classified as normal, hyperplastic polyp, non‐advanced adenoma, advanced adenoma, or colorectal cancer. ^b^ *n* = 124 participants had multiple surveillance colonoscopies during the audit period.

There were 1494 colonoscopies in participants 75 years and older during the audit period. Colonoscopies undertaken for reasons other than surveillance were excluded (364 for symptoms, 216 for follow‐up of a positive FOBT result and 97 for follow‐up of an incomplete procedure). A further 52 colonoscopies were excluded from the analysis as they were in participants with inflammatory bowel disease, poor bowel preparation or had indeterminate pathology outcomes. The final number of surveillance colonoscopies included in the data analysis was 698 (Fig. 1), which were from 574 unique participants.

The demographics associated with the 698 surveillance colonoscopies assessed are summarized in Table 1. These included procedures in 55.6% males, with a median age of 78.5 years (IQR 76.6–80.8). 67.3% of the procedures were done in the age group 75–79.9 years, 28.4% in the age group 80–84.9 years, with 4.3% completed in individuals over 85 years. There were 686 procedures completed in individuals who were able to be classified with a CCI, which included a majority with a CCI of ≥5. 27.5% of colonoscopies were in participants with a prior history of CRC in their lifetime. Almost half of the colonoscopies occurred after an advanced neoplasia, with 10% having CRC diagnosed and 39.1% having an advanced adenoma at the immediate prior colonoscopy (Table 1).

**Table 1:** Demographics of entire cohort undergoing surveillance colonoscopy aged 75 years and older, and the cohort with lifetime CRC.

	Entire cohort (*n* = 698, 100.0%)		Lifetime CRC cohort	
			(*n* = 192, 27.5%)	
	*n*	%	*n*	%
Age categories (years)				
75–79	474	67.9	111	57.8
80–84	194	27.8	64	33.3
≥85	30	4.3	17	8.8
Age (median, IQR)	78.45 (76.66, 80.76)		79.37 (77.31, 82.28)	
Sex				
Female	310	44.4	70	36.5
Male	388	55.6	122	63.5
Lifetime CRC				
No	506	72.3		
Yes	192	27.5		
CRC at index colonoscopy				
No CRC at index	527	75.50		
CRC at index	171	24.50		
Years since index procedure (median IQR)	6.10 (3.10, 11.31)		4.12 (1.25, 10.47)	
Findings at prior colonoscopy				
Non‐advanced	355	35.8	91	47.4
Advanced adenoma	273	39.1	31	16.2
CRC at prior colonoscopy	70	10.0	70	34.5
Years since immediate prior colonoscopy (median (IQR)	3.13 (1.13, 3.13)		1.37 (1.00, 3.04)	
Family history CRC				
No	486	69.6	141	73.4
Yes	212	30.37	51	26.6
Body mass index (median, IQR) (*n* = 499)	27.34 (24.51, 30.84)		26.84 (24.76, 30.47)	
Smoking (*n* = 583)				
Nonsmoker	295	50.6	73	45.9
Ex‐smoker	244	41.9	74	46.5
Smoker	44	7.5	12	7.5
History of alcohol intake (*n* = 440)				
Non‐drinkers	311	71.8	80	69.6
Drinkers	122	28.2	35	30.4
Polypharmacy (*n* = 690)				
<5 medications	356	51.6	102	54.5
≥5 medications	334	48.4	85	45.5
Socioeconomic position^†^				
Low	279	40.0	76	39.6
High	419	60.0	116	60.4
Charlson Comorbidity Index mean (SD) (*n* = 686)	5.28 (±1.63)		6.48 (±1.32)	
Number of previous colonoscopies				
1	234	33.5	68	35.4
2	148	21.2	44	22.9
3	108	15.5	21	10.9
≥4+	208	29.8	59	30.75
Number of previous colonoscopies, median (IQR)	2 (1, 4)		2 (1, 4)	

^†^ Socioeconomic position was categorized based on the deciles from the Index of Relative Socioeconomic Advantage and Disadvantage where deciles 1–5 considered low and 6–10 as high.

CRC, colorectal cancer; IQR, inter quartile range.

### Incidence and risk factors for CRC at surveillance colonoscopy

The incidence of CRC in surveillance colonoscopies in participants over 75 years was 1.58% (*n* = 11/698), where the incidence was higher in participants with prior history of CRC (8/129, 4.2%) and lower in participants without prior CRC (3/569, 0.5%). The majority of cancer findings (*n* = 8/11) were metachronous or recurrent CRC following a prior CRC diagnosis. Of the three cases that had not had prior CRC, two participants had a recent advanced adenoma finding, all were males aged between 79.0 and 80.2 years, and two had a family history of CRC. Among the 11 CRC cases, two were in stage I, three in stage II, three in stage III, and three in stage IV.

Based on the Poisson regression analysis, age ≥85 years (IRR 5.76, 95% CI 1.59–20.86), a history of CRC (IRR 5.88, 95% CI 1.51–22.85) and active smoking (IRR 4.89, 95% CI 1.00–24.39) were independently associated with diagnosis of CRC at surveillance colonoscopy. The development of CRC was not significantly associated with sex, socioeconomic position, CCI, BMI, alcohol intake status, polypharmacy, or a family history of CRC (*P* > 0.05; Table 2).

**Table 2:** Variables associated with a finding of colorectal cancer at surveillance colonoscopy

	Whole cohort (*n* = 698)		Lifetime CRC cohort (*n* = 192)	
	Adjusted incidence rate ratio (95% CI)^a^	*P* value	Adjusted incidence rate ratio (95% CI)^a^	*P* value
Age category (years)				
75–79	Reference		Reference	
80–84	0.64 (0.12–3.30)	0.59	0.44 (0.06–3.43)	0.43
≥85	5.76 (1.59–20.86)	0.01	7.69 (1.51–39.22)	0.01
Sex				
Female	Reference		Reference	
Male	1.02 (0.28, 3.72)	0.97	0.52 (0.11, 2.48)	0.41
Lifetime CRC				
No	Reference		‐	‐
Yes	5.88 (1.51–22.85)	0.01	‐	‐
Family history of CRC				
No	Reference		Reference	
Yes	0.65 (0.14, 3.01)	0.58	0.57 (0.06, 5.11)	0.62
Smoking status				
Nonsmoker	Reference		Reference	
Ex‐smoker	0.59 (0.15–3.63)	0.48	0.69 (0.13–3.33)	0.69
Active smoker	4.89 (1.00–24.39)	0.05	11.60 (1.53–87.79)	0.02

CI, confidence interval; CRC, colorectal cancer.

^a^ The significant variables remained the same where the model was adjusted for age, sex, socioeconomic position, lifetime CRC (only for the whole cohort), Charlson comorbidity index, body mass index, smoking, and history of alcohol intake.

When limiting the cohort to those who had a lifetime history of CRC (n = 192), it was found that age ≥85 years (IRR 7.69, 95% CI 1.51–39.22) and active smoking status (IRR 11.60, 95% CI 1.53–87.79) remained significantly associated with CRC at surveillance colonoscopy (Table 2).

### Incidence and risk for advanced adenoma at surveillance colonoscopy

More than a third (37.85%; *n* = 260/698) of the surveillance colonoscopies in participants over 75 years had a finding of advanced adenoma. Multivariable logistic regression revealed that having advanced adenoma at the most recent prior colonoscopy (IRR 1.61, 95% CI 1.31–1.97) and presence of polypharmacy (IRR 1.24, 95% CI 1.01–1.53) were significantly associated with a finding of advanced adenoma at surveillance colonoscopy (Table 3). Age, sex, socioeconomic position, family history of CRC, CRC at index colonoscopy, CCI, alcohol intake status, smoking status and BMI were not significantly associated with a finding of advanced adenoma (*P* > 0.05; Table 3).

**Table 3:** Variables associated with a finding of advanced adenoma at surveillance colonoscopy

	Whole cohort (*n* = 687)		Lifetime CRC cohort (*n* = 181)	
	Adjusted incidence rate ratio (95% CI)^a^	*P* value	Adjusted incidence rate ratio (95% CI)^a^	*P* value
Age category (years)				
75–79	Reference		Reference	
80–84	1.11 (0.85, 1.32)	0.58	0.73 (0.40, 1.34)	0.31
≥85	0.85 (0.49, 1.46)	0.56	0.49 (0.18, 1.37)	0.17
Sex				
Female	Reference		Reference	
Male	1.17 (0.95, 1.44)	0.14	1.44 (0.87, 2.38)	0.15
Socioeconomic position				
Low	Reference		Reference	
High	0.90 (0.75, 1.09)	0.29	0.71 (0.45, 1.12)	0.14
CRC at index colonoscopy				
No	Reference		Reference	
Yes	0.79 (0.58, 1.08)	0.14	0.84 (0.43, 1.63)	0.61
Finding at previous colonoscopy				
Non‐advanced findings	Reference		Reference	
Advanced adenoma	1.61 (1.31, 1.97)	<0.001	2.19 (1.29–3.72)	0.004
CRC	1.27 (0.81, 1.98)	0.30	1.31 (0.76, 2.27)	0.33
Family history CRC				
No	Reference		Reference	
Yes	0.91 (0.73, 1.14)	0.41	0.71 (0.40, 1.25)	0.23
Smoking				
Nonsmoker	Reference			
Ex‐smoker	0.96 (0.77, 1.21)	0.74	0.96 (0.63, 1.49)	0.87
Active smoker	0.89 (0.60, 1.34)	0.60	0.33 (0.10, 1.72)	0.1984
Body mass index (kg/m^2^)	1.01 (0.99, 1.03)	0.29	1.02 (0.96, 1.09)	0.44
Polypharmacy				
<5 medications	Reference		Reference	
≥5 medications	1.24 (1.01, 1.53)	0.04	1.40 (0.79, 2.48)	0.25
Charlson Comorbidity Index	1.01 (0.93, 1.08)	0.87	1.05 (0.83, 1.33)	0.67
History of alcohol intake				
Non‐drinkers	Reference		Reference	
Drinkers	0.95 (0.75, 1.21)	0.68	1.37 (0.77, 2.45)	0.28

CRC, colorectal cancer.

^a^ The models were adjusted for age, sex, socioeconomic position, CRC at index colonoscopy, finding at previous colonoscopy, CRC at index, family history of CRC, smoking, body mass index, polypharmacy, Charlson Comorbidity Index, and history of alcohol intake.

In individuals with a lifetime history of CRC, the only variable significantly associated with advanced adenoma at surveillance was prior advanced adenoma (IRR 2.19, 95% CI 1.29–3.72).

## Discussion

Advancing age is a well‐known risk factor for development of advanced adenoma and subsequent CRC risk.^4^, ^26^, ^27^, ^28^, ^29^, ^30^, ^31^, ^32^, ^33^, ^34^, ^35^ Current guidelines lack clear direction on managing surveillance in older cohorts. This study found advanced neoplasia (CRC or advanced adenoma) in more than one third of the older (≥75 years of age) surveillance population. A finding of advanced adenoma at immediate previous colonoscopy, age ≥85 years, active smoking, polypharmacy, and a previous history of CRC were associated with advanced neoplasia in this older population. Of note, most participants within this cohort, continued surveillance due to a personal history of CRC, and these individuals were identified at highest risk of CRC among those who continued surveillance beyond 75 years.

The Australian National Cancer Control Indicators reported CRC incidence as threefold higher at 389/100000 in the ≥75 year age group, as compared to 130/100000 in the 50–74 years age group.^27^ We found advanced neoplasia in more than a third (37.85% advanced adenoma and 1.58% CRC) of surveillance participants over 75 years. This is higher when compared to a finding of 13.1% advanced neoplasia in younger adults (mean age 63 years), in the same South Australian surveillance program.^36^ When comparing a finding of CRC alone, our incidence was similar in participants without previous CRC to that of the younger adults cohort (1.1%),^36^ but much higher in those with lifetime CRC in older cohort (4.2%). Our findings highlight the importance of regular surveillance colonoscopies for individuals with a lifetime history of CRC as well as advanced findings in their immediate prior colonoscopies, regardless of age. We observed equal distribution of advanced‐stage (Stages III and IV) and earlier stage (Stages I and II) CRC which may be attributed to the finding of higher metachronous or recurrent cancers in the elderly people following a prior CRC diagnosis, necessitating frequent colonoscopy follow‐up. Thus, it is important to identify individuals with higher risk of CRC who are ≥75 years, and those fit to undergo colonoscopy.^37^

One concern in this older population is an increased risk of procedural complications, where age and increasing comorbid state are known to be associated with an increased risk of hospitalization and patient mortality.^4^, ^16^, ^29^, ^30^, ^32^, ^38^ Age ≥75 years and increasing CCI are both known risk factors for adverse colonoscopy outcomes.^29^ Another study by Causada‐Calo et al. also investigated 30‐day postoperative complications in ≥75 and 50–74 year age groups, finding a higher incidence of complications in the ≥75 group (6.8%) compared to the younger cohort (2.6%).^32^ In an average risk population, colonoscopy had no benefit in CRC mortality in participants over 75 years with more than three comorbidities.^39^ In our study, increasing CCI was not associated with advanced adenoma or CRC, but our cohort was biased toward individuals that the proceduralists considered suitable for surveillance colonoscopy, and details on postoperative complications were not collected. However, considering the association between increasing CCI and post‐procedural complications, CCI may be a useful tool in justifying cessation of surveillance colonoscopy. Thus, increasing comorbidities could be considered a valid reason to discontinue surveillance, particularly in cases of advanced adenoma only, which may take years to progress to CRC.

It was, however, observed that more individuals having lifetime CRC had a CCI of 5 or more and were older when compared with their counterparts without lifetime CRC. This is an important and relevant finding, considering the above finding that older individuals with lifetime CRC were found to be at increased risk of further CRC. Exact procedural risk and overall prognosis needs to be considered in these individuals when making decision on continuing surveillance with the colonoscopy. There is relative variability in the health and life expectancy of older people,^15^, ^40^ and it is therefore not recommended for age to be a solitary deciding factor in surveillance decisions.^26^, ^27^, ^28^, ^31^, ^41^ Objective consideration of these factors,^42^ along with patient choice, should be incorporated into decision making regarding ongoing surveillance. We assessed the literature for existing instruments to assist in such decision making. The ePrognosis CRC Screening Survey provides personalized recommendations for CRC screening based on age, comorbid and functional status and participants' self‐assessment of their health.^33^, ^43^ Of note, this tool does not consider baseline CRC risk and previous surveillance results, and assumes participants accurately assess their own health. There is a known poor correlation between self‐reported health and actual predicted life expectancy in older participants.^44^ Another study found that in participants with a twofold increased CRC risk and without moderate comorbidities, screening benefit outweighed harm until age 84 years.^33^ Both these instruments were studied in relation to screening colonoscopies, and therefore do not apply in the surveillance setting. Further investigation of prognostic factors following surveillance colonoscopies and the application of existing or novel risk calculators in participants over 75 years need to be undertaken.

Although it was not associated with CRC, we found that polypharmacy was an independent risk factor of advanced adenoma. This is therefore another factor that could be included in risk calculators. Furthermore, instead of continuing colonoscopies, addressing the underlying health issues, as well as polypharmacy itself, may have a positive impact on preventing pre‐cancerous neoplasia, as well as quality of life and overall survival.^45^

While this study contributes to the small pool of Australian data (*n* = 698) on this topic, we acknowledge its limitations. Our study population included only participants at elevated risk for CRC who had surveillance colonoscopy beyond 75 years and excluded those who had been removed from surveillance previously. This population is thus participants in whom patients and treating clinicians collectively felt it necessary to continue surveillance, potentially causing overestimation of incidence and strength of associated risk factors for advanced adenoma or CRC. Also, this study did not assess the yield of colonoscopies undertaken for reasons other than surveillance. However, this will be an important future consideration to determine whether these colonoscopies are also warranted in the over 75 years cohort. Another limitation arose during data collection, with some records only partially completed, most notably for smoking status and alcohol intake, however, the multivariable regression analyses were conducted on the imputed dataset to get a more precise estimate on the associations between those incomplete variables and advanced adenoma or CRC risk. Future multicentre larger prospectives studies that considers a more comprehensive risk factors such as dietary habits and genetic risk scores are warranted. Furthermore, the impact of COVID‐19 on the uptake of surveillance colonoscopy may have impacted the incidence of advanced neoplasia in the final year of the audit (2020), given the delay in surveillance, as found in a recent study in South Australia.^46^

## Conclusions

A significant incidence of advanced neoplasia was found in surveillance colonoscopy participants over 75 years of age. In this population, a higher incidence of CRC was found in participants who had a personal history of CRC, were active smokers, and were aged ≥85 years (most of whom also had a personal history of CRC). Meanwhile, the risk for advanced adenoma was elevated in those individuals taking more than five medications (polypharmacy), and who had advanced adenoma at their prior colonoscopy. Whether this older population will have extension to or improved quality of life with treatment, requires further longitudinal research. Greater emphasis should be placed on patient health, number and type of medications and comorbidities when making surveillance decisions. A validated personalized analysis tool for predicting appropriateness of surveillance would be a useful guide for participants and clinicians.
